# Laryngopharyngeal Mucosal Injury Due to Nasogastric Tube Insertion during Cardiopulmonary Resuscitation: A Retrospective Cohort Study

**DOI:** 10.3390/jcm13010261

**Published:** 2024-01-02

**Authors:** Kazuyuki Miyamoto, Hiromi Takayasu, Shino Katsuki, Atsuo Maeda, Keisuke Suzuki, Motoyasu Nakamura, Noriko Hida, Takehiko Sambe, Masaharu Yagi, Jun Sasaki, Munetaka Hayashi, Kenji Dohi

**Affiliations:** 1 Department of Emergency, Critical care and Disaster Medicine, School of Medicine, Showa University, Hatanodai, Shinagawa-ku, Tokyo 1428666, Japan; rinomizuki@yahoo.co.jp (H.T.); katsukishinok@gmail.com (S.K.); atsuo@med.showa-u.ac.jp (A.M.); k.s.07202251@gmail.com (K.S.); motoyasu@med.showa-u.ac.jp (M.N.); masaharuy0130@gmail.com (M.Y.); jun-sa@da2.so-net.ne.jp (J.S.); munetaka@med.showa-u.ac.jp (M.H.); kdop@med.showa-u.ac.jp (K.D.); 2 Department of Emergency, Critical care and Disaster Medicine, Showa University Fujigaoka Hospital, Fujigaoka Aoba-ku, Yokohama City 2278501, Japan; 3 Department of Clinical Pharmacy, Division of Clinical Research and Development, School of Pharmacy, Showa University, Kita-karasuyama, Setagaya-ku, Tokyo 1578577, Japan; n.hida@med.showa-u.ac.jp; 4 Department of Pharmacology, Clinical Pharmacology, School of Medicine, Showa University, Hatanodai, Shinagawa-ku, Tokyo 1428666, Japan; t-sambe@med.showa-u.ac.jp

**Keywords:** cardiopulmonary resuscitation, nasogastric tube insertion, laryngopharyngeal mucosal injury, video laryngoscope, cardiopulmonary arrest, insertion time, insertion number

## Abstract

Background: Patients under cardiopulmonary resuscitation (CPR) are at high risk of aspirating gastric contents. Nasogastric tube insertion (NGTI) after tracheal intubation is usually performed blindly. This sometimes causes laryngopharyngeal mucosal injury (LPMI), leading to severe bleeding. This study clarified the incidence of LPMI due to blind NGTI during CPR. Methods: We retrospectively analyzed 84 patients presenting with cardiopulmonary arrest on arrival, categorized them into a Smooth group (Smooth; blind NGTI was possible within 2 min), and Difficult group (blind NGTI was not possible), and consequently performed video laryngoscope-assisted NGTI. The laryngopharyngeal mucosal condition was recorded using video laryngoscope. Success rates and insertion time for the Smooth group were calculated. Insertion number and LPMI scores were compared between the groups. Each regression line of outcome measurements was obtained using simple regression analysis. We also analyzed the causes of the Difficult group, using recorded video laryngoscope-assisted videos. Results: The success rate was 78.6% (66/84). NGTI time was 48.8 ± 4.0 s in the Smooth group. Insertion number and injury scores in the Smooth group were significantly lower than those in the Difficult group. The severity of LPMI increased with NGT insertion time and insertion number. Conclusions: Whenever blind NGTI is difficult, switching to other methods is essential to prevent unnecessary persistence.

## 1. Introduction

**Figure 1:**
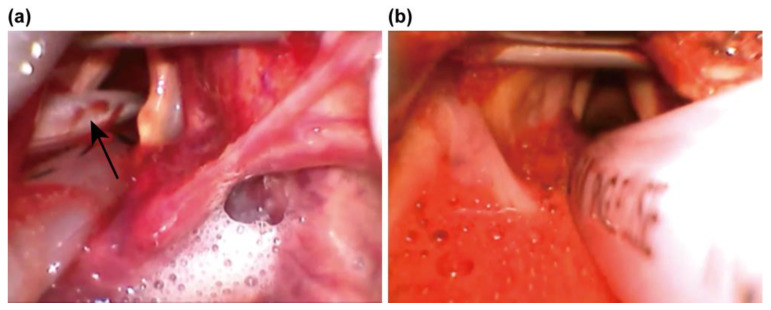
A schematic illustrating the complications of nasogastric tube insertion. (**a**) Video laryngoscopy showing that the nasogastric tube (arrow) is placed in the trachea with the intubation tube; (**b**) Severe bleeding from the site of mucosal injury during nasogastric tube insertion after initiation of anticoagulation therapy.

The estimated prevalence of non-traumatic out-of-hospital cardiac arrests in the United States, across all age groups, is 356,461, with only 10% of patients being discharged after treatment [1]. Even for those discharged, complications in relation to consciousness and cognitive deficits are often suffered, as the brain is vulnerable to hypoxia. Hence, treatments for cardiac arrest need to be administered promptly, as they are most effective when provided early. In some cases, the introduction of extracorporeal life support (ECLS), to provide a circulation of oxygenated blood to improve tissue perfusion while continuing resuscitation, is necessary [2]. Resuscitation at the emergency department (ED) requires several procedures to be performed within a limited time period and with limited assistance, to minimize complications. Unlike patients scheduled for surgery, who are fasting, patients under CPR are at high risk of aspirating gastric contents [3,4]. Moreover, the rapid increase in intrathoracic pressure with chest compression and the retention of air in the stomach, due to external ventilation, increase the risk of vomiting [5]. When vomiting and regurgitation occur, gastric contents can enter the trachea. This is more prone to occur in the supine position, and aspiration of gastric contents cannot be completely prevented, even with intubation [6]. Nasogastric tube (NGT) insertion remains the easiest and best technique for accessing the gastrointestinal tract [7]. Therefore, placing an NGT after tracheal intubation during CPR is common. The 16-French Salem Sump^TM^ tube (Covidien, Mansfield, MA, USA), which is relatively rigid, is widely used, as it is easy to insert blindly. It is introduced into one of the nares blindly, then passed through the esophagus into the stomach. The placement is usually easy; however, in some cases, the insertion is difficult, as there is a physiological stenosis site at the nasal cavity, pharynx and esophagus. Moreover, head flexion and the endotracheal tube could facilitate the insertion in a difficult case [7,8]. Indeed, there have been reports of inadvertent insertion of the NGT into the lung (Figure 1a), even in intubated patients [9], and some cases of mucosal injury of the laryngopharynx and the gastrointestinal tract caused by the NGT. Recently, the number of older patients undergoing anticoagulation and antiplatelet therapy for atrial fibrillation, pulmonary embolism, stroke, and acute coronary syndrome (ACS) has been increasing, owing to the aging of the population [10]. Furthermore, post-resuscitation patients often require anticoagulation therapy, due to acute coronary syndrome, pulmonary embolism, or the introduction of cardiac or pulmonary extracorporeal membrane oxygenation. In those patients with bleeding diathesis, minor trauma to the laryngopharynx resulting from blind NGT insertion can cause severe bleeding, which may be difficult to arrest (Figure 1b). In the previous study, there were some reports that blind NGT insertion in intubated patients injured hypopharynx, trachea and esophageal mucosa [11,12]. However, the incidence of laryngopharyngeal mucosal injury due to blind NGT insertion during CPR is poorly understood.

A video laryngoscope (VLS), endowed with a miniature video camera, enables the operator to visualize the glottis indirectly [13]. This technology was introduced in the 2000s. Video laryngoscopy improves the Cormack–Lehane grade, and achieves the same or higher intubation success rate with less duration, compared to direct laryngoscopy [14,15]. A VLS also enables visualization of the glottis, pharynx, and esophageal inlet.

This retrospective study aimed to clarify the incidence of laryngopharyngeal mucosal injury due to blind NGT insertion during CPR. Moreover, we also analyzed the causes for which blind NGT insertion could not be performed.

## 2. Materials and Methods

### 2.1. Study Design and Participants

**Figure 2:**
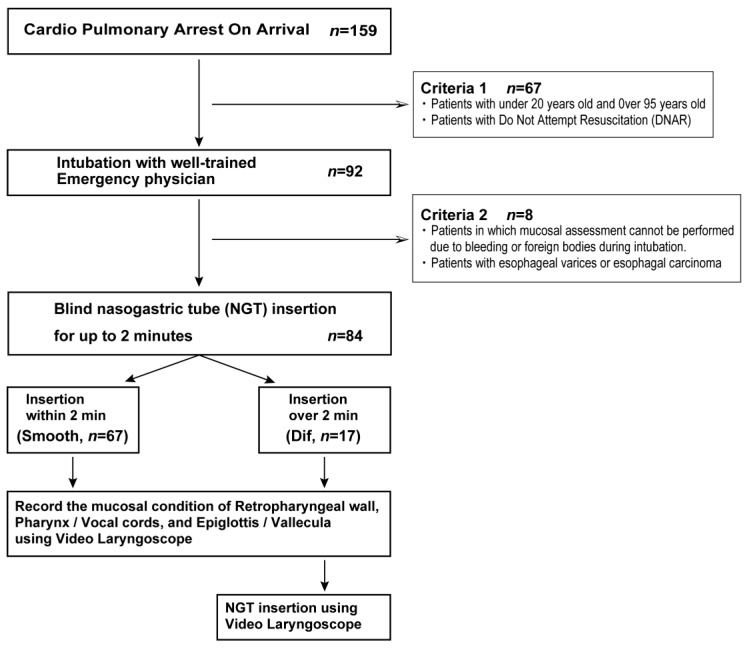
Study protocol. One hundred and fifty-nine patients with cardiopulmonary arrest on arrival were referred to our emergency department. Of these, 67 patients were excluded from this study based on criterion 1, and eight were excluded based on criterion 2. Finally, 84 patients (Smooth group: *n* = 67 and Difficult group: *n* = 17) were retrospectively analyzed in this study.

The present study was performed retrospectively, using nursing records and videos recorded with a VLS stored on a university computer. All human research protocols were approved and supervised by the Clinical Trial Review Board of Showa University (#2023-060-A), and adhered to the Council for International Organizations of Medical Sciences Ethical Guidelines for Biomedical Research. Written informed consent was obtained from all participants and/or their legal guardians. The study has been performed in accordance with the tenets of the Declaration of Helsinki. The study was conducted between April 2017 and March 2020, and included patients with cardiopulmonary arrest on arrival (CPOA), who were referred to the ED at Showa University Fujigaoka Hospital (Figure 2). We accessed the data from 17 July 2023 to 21 July 2023 for research. However, we did not have access to patient-identifying information. Patients < 20 years and >95 years of age or patients who were issued with a “Do Not Attempt Resuscitation” order were excluded from this study (exclusion criterion 1). NGTs were not inserted in patients with bleeding such as hematemesis or the presence of foreign bodies, or in patients with esophageal varices or carcinoma (exclusion criterion 2).

### 2.2. Protocol

Resuscitation was performed according to the Japan Resuscitation Council Resuscitation Guidelines 2015. Paramedics transferred patients to the ED with continuing manual chest compressions and laryngeal mask airway (LMA) or mask ventilation. After arrival, a non-invasive cardiac support pump (AutoPulse^®^, ZOLL, Chelmsford, MA, USA) was set up, and an intravenous catheter was rapidly inserted. An experienced emergency physician removed the LMA and intubated (7.0 mm Portex^TM^ Tracheal Tube, Smith Medical, Ashford, Kent, UK, with 8–10 mL cuff air), using a direct laryngoscope or VLS (C-MAC^®^ system, KARL STORZ, Tuttlingen, Germany), with continuing chest compression. After intubation, an NGT with a 16-French catheter scale (Salem Sump^TM^ tube, Covidien, Mansfield, MA, USA) made of polyvinyl chloride was blindly inserted with lubricant (Hydroxyethyl cellulose and glycerin) to decompress the air and stomach contents during chest compression in all patients. In our institution, there is a rule of a maximum of 2 min for blind NGT insertion during resuscitation. In patients with NGTs that were not inserted blindly within 2 min, insertion was temporarily interrupted. Subsequently, the NGT was inserted under VLS assistance. The condition of the laryngopharyngeal (retropharyngeal wall (RPW), pharynx (Px)/vocal cords (VC), and epiglottis (Eg)/vallecula (VL)) mucosa was recorded using the VLS, for all patients. We defined the patients in whom the NGT could be inserted within 2 min as the Smooth group (Smooth). In contrast, the patients in whom the NGT was not inserted blindly within 2 min were defined as the Difficult group (Dif) (Figure 2). All procedures were performed by four well-trained senior emergency physicians, with more than 10 years of experience. They were familiar with blind or VLS-assisted NGT insertions and intubations using video or direct laryngoscope. During these procedures, other emergency physicians were recording medical history and conducting a physical examination and echocardiography, to determine the cause of cardiac arrest.

### 2.3. NGT Insertion

Prior to NGT insertion, the tip of the NGT was placed on the patient’s xiphoid, and the length from the xiphoid to the earlobe was measured, assuming that it was sufficiently long to reach the stomach. The NGT was inserted blindly, with lubricant gel (3–4 mL), from the right nasal cavity. If the right nasal cavity was narrow, insertion was made through the left cavity. For patients in whom the NGT could not be inserted blindly within 2 min, the insertion was temporarily interrupted. Then, the NGT was inserted while observing the tip of the tube, using a VLS. When the NGT tip did not go to the esophageal inlet, we twisted the tube, with neck rotation or external manipulation, and led the tip to the esophageal inlet. In our study, we did not use the Magil forceps or other instruments to insert the NGT. We defined successful NGT insertion as an insertion at least as long as the predicted length, and radiographic confirmation of its tip at the gastric fundus was seen on a radiograph. The radiographic interpretation was confirmed by several physicians.

### 2.4. Definitions of NGT Insertion Time and Insertion Number

NGT insertion time was defined as the time required for the emergency physician to insert an NGT, confirm the auscultation over the epigastrium during air injection, and confirm that the tube was properly inserted. The NGT insertion number was defined as the number of times the physician pulled back the NGT after perceiving resistance during insertion. These data were referred to from nursing records.

### 2.5. Evaluation of Laryngopharyngeal Mucosal Injury after NGT Insertion

**Figure 3:**
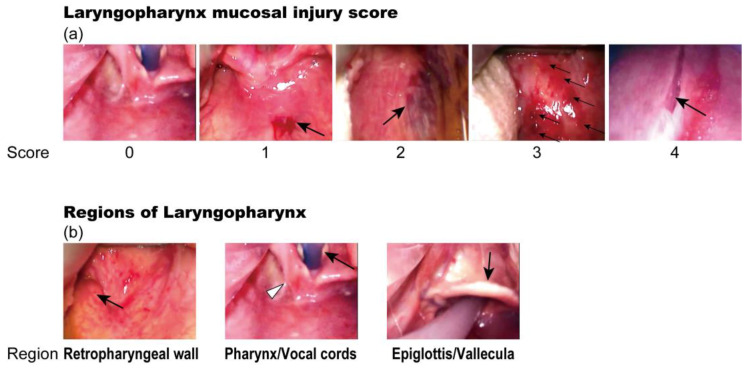
Evaluation of laryngopharyngeal mucosal injury after nasogastric tube insertion. (**a**) Laryngopharyngeal mucosal injury was evaluated using injury scores: 0, no injury; 1, erythema (arrow); 2, hematoma (arrow); 3, more than 10 spots of erythema and hematoma (arrows); and 4, laceration (arrow); (**b**) The three regions of the laryngopharynx (retropharyngeal wall (arrow indicates the uvula), pharynx (white arrowhead indicates the hypopharynx, and black arrow indicates the vocal cord), and epiglottis/vallecula (arrow indicates the epiglottis)) were evaluated separately. The highest score for each region was defined as the injury score for that region. The sum of the injury scores of the three regions was used as the score for the patient.

Laryngopharyngeal mucosal injury after NGT insertion was evaluated using injury scores (Figure 3a; 0: no injury, 1: erythema, 2: hematoma, 3: more than 10 spots of erythema and hematoma, and 4: laceration) [16]. The three parts of the laryngopharynx (RPW, Po/VC, and Eg/VL) were evaluated separately (Figure 3b). The highest score for each region was defined as the injury score for that region. The sum of the injury scores from the three regions was defined as the total score for the patient. Two investigators (K.S. and M.N.), who were blinded to the experimental groups, performed the scoring independently, while watching the anonymized videos of the VLS, and the average values of their scores were calculated.

### 2.6. Outcome Measure

The success rate of blind NGT insertion within 2 min in the Smooth group and NGT insertion under VLS assistance in the Dif group were evaluated. The insertion time of the Smooth and Dif groups, in which the NGT was inserted under the assistance of a VLS, were evaluated. Patient background, number of NGT insertions within 2 min, and injury scores (total and region-specific) were compared between Smooth and Dif groups. Furthermore, correlations between injury score, age, insertion time, and insertion number were examined. Subsequently, the regression line was generated, using a simple regression analysis.

### 2.7. Video Analysis of the Causes of Blind NGT Insertion in Dif Group

The videos of the Dif group were analyzed to detect the cause of failed blind NGT insertion. Moreover, we analyzed the cases in which the NGT could not be inserted under VLS assistance. Two investigators (K.S. and M.N.), who were blinded to the experimental groups, watched the anonymized video and evaluated the cause together.

### 2.8. Statistical Analysis

All statistical analyses were performed using JMP Pro version 16 (SAS, Madison, WI, USA). Data are reported as mean ± standard error of the mean or median (interquartile range) Student’s t-test or Mann–Whitney U test was used to determine the significance of differences between the two groups. The Steel–Dwass test was used for multiple comparisons. All statistical tests were two-tailed, and a *p*-value < 0.05 was considered statistically significant. Pearson’s correlation coefficient values were used for correlation analyses. Subsequently, a simple regression analysis was performed, and a regression line was obtained.

## 3. Results

### 3.1. Patient Characteristics

One hundred and fifty-nine patients with CPOA were referred to our ED. Of these, 67 patients were excluded from this study based on criterion 1, and eight patients were excluded based on criterion 2. Finally, 84 patients were retrospectively analyzed (Figure 2). The Smooth and Dif groups included 67 and 17 patients, respectively. The Smooth group included 35 men and 32 women, and the Dif group included 13 men and 4 women. The average age of patients in the Smooth and Dif groups was 77 (61.5–85)and 77 (71–84) years, respectively (Table 1). There were no significant differences between the two groups based on sex or age.

### 3.2. Success Rate, Insertion Time, and NGT Insertion Number

The success rate in blind NGT insertion within 2 min during resuscitation was 78.6% (66/84). In 67 patients, the NGT was inserted smoothly (Smooth group) within 2 min, and there was one patient in whom the NGT was inadvertently placed in the trachea (success rate, 66/67, 98.5%). There were 17 patients in whom the NGT could not be inserted within 2 min (Dif group). In the Smooth group, the time taken for NGT insertion was 48.8 ± 4.0 s. In contrast, the success rate of VLS-assisted NGT insertion in the Dif group was 76.5% (13/17) and the insertion time was 54.8 ± 3.0 s after switching to VLS-assisted insertion. The NGT insertion number was significantly increased in the Dif group compared with that in the Smooth group (Smooth, 1 (1–2) and Dif, 8 (6–11); Mann–Whitney U test, *p* < 0.01) (Table 1).

### 3.3. Blind NGT Insertions over 2 Min Increased Mucosal Injury, Especially at the RPW

The total injury score was significantly lower in the Smooth group than that in the Dif group (Smooth, 0 (0–1) and Dif, 7 (4–8); Mann–Whitney U test, *p* < 0.01). A region-specific examination also revealed that the injury score was significantly lower in the Smooth group than that in the Dif group (RPW: Smooth, 0 (0–1) and Dif, 2 (2–4); Mann–Whitney U test, *p* < 0.01 vs. Px/VC: Smooth, 0 (0–1) and Dif, 2 (1–3); Mann–Whitney U test, *p* < 0.01 vs. Eg/VL: Smooth, 0 (0) and Dif, 2 (0–3); Mann–Whitney U test, *p* < 0.01; Table 2). In both groups, laryngopharyngeal mucosal injury with NGT insertion was most severe in the RPW, which had a significantly higher injury score than Eg/VL (Smooth: RPW, 0 (0–1) and Eg/VL, 0 (0); Steel–Dwass test, *p* < 0.05 vs. Dif: RPW, 2 (2–4) and Eg/VL, 2 (0–3); Steel–Dwass test, *p* < 0.05).

### 3.4. Correlations among NGT Insertion Number, Insertion Time, and Injury Scores

**Figure 4:**
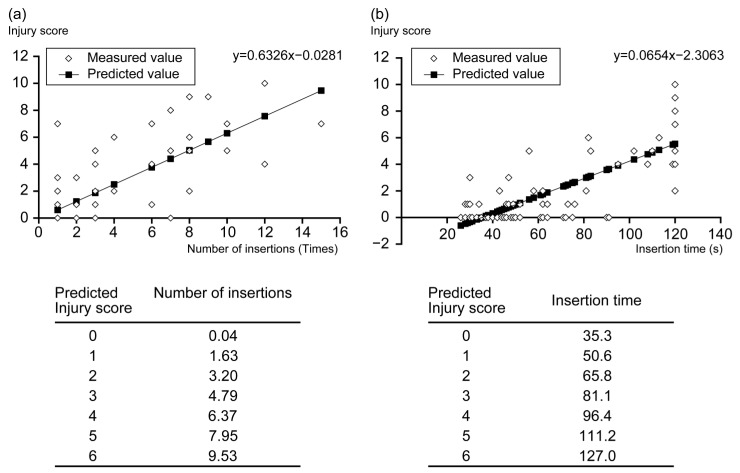
Regression lines for the insertion number, insertion time, and injury score. Regression lines are helpful in predicting the injury score (*p* < 0.05). (**a**,**b**) These regression lines suggested that the predicted injury score was low in cases where the nasogastric tube could be inserted blindly within a short duration and with a lower insertion number.

There were strong positive correlations between insertion number and injury score (*r* = 0.76), insertion time and injury score (*r* = 0.79), and insertion number and insertion time (*r* = 0.76) (Table 3). We performed a simple regression analysis to elucidate the relationship between insertion number and injury score and between insertion time and injury score. In this analysis, the regression lines were useful in predicting the injury score (*p* < 0.05). These regression lines suggested that the predicted injury score was low in cases where the NGT could be inserted within a short duration and with a smaller number of insertions (Figure 4a,b).

### 3.5. Why Was the Blind NGT Insertion Not Possible?

In 17 patients, NGT insertion was performed under the VLS-assisted protocol, as it could not be inserted blindly within 2 min (Dif group). The success rate of VLS-assisted NGT insertion in the Dif group was 76.5% (13/17). In 10 out of 17 patients (58.8%), the NGT advanced smoothly with the assistance of a VLS. In three patients (17.6%, 3/17), the NGT always advanced toward the trachea (Video S1); therefore, we twisted the tube, with neck rotation or external manipulation, and led the tip to the piriform sinuses. Moreover, in four patients (23.5%, 4/17), the tip of the NGT could be advanced toward the piriform sinuses, but could not be passed into the esophageal inlet (Video S2). Therefore, we adjusted the angle of insertion with neck rotation or external manipulation, and maneuvered the tube into the esophageal inlet. In all 17 patients, the NGT was maneuvered through the esophageal inlet into the upper esophagus with the assistance of a VLS. However, the procedure was discontinued in four patients (23.5%, 4/17), due to perceived resistance in the middle or lower esophagus during the advancement of the tube (Video S3). During VLS-assisted insertion of the NGT, we noticed that when we pushed down an NGT that was stuck in the middle or lower esophagus a sharp corner was created whenever the NGT was bent. These sharp corners attached to the mucous membranes of the RPW (Video S4).

## 4. Discussion

An NGT is a flexible double- or single-lumen tube, which is made of polyvinyl chloride, polyurethane, or silicone. Most NGTs are usually inserted blindly, with the patient in a seated position at the bedside [8]. In patients who are awake, the procedure is not difficult, as patients can cooperate by swallowing and can report abnormalities if they occur. On the other hand, in intubated patients, NGT insertion can be difficult. Ozer and Benumof [11] have reported that gastric tube insertion failed in 23% of intubated patient under general anesthesia. In our study, in 17 of 84 (20.2%) patients, NGTs could not be inserted blindly within 2 min, which was similar to their results. In contrast, in 67 of 84 (79.8%) patients, NGTs could be inserted blindly and smoothly, and the time required for insertion was 48.8 ± 4.0 s. Our results indicate that blind NGT insertion during CPR is smooth and rapid in most patients. However, NGT cannot be inserted blindly in a certain number of patients.

A cause of difficulty is the passage of the gastric tube in intubated patients under general anesthesia, and Ozer and Benumof [11] have evaluated it at the laryngeal level, using a fiberscope. They reported that the impaction sites were the piriform sinuses (46%), arytenoid cartilages (25%), and trachea (21%). We inserted the NGT under VLS assistance, and the recorded videos were analyzed retrospectively. NGTs were placed smoothly in 10 patients (58.8%, 10/17), as the esophageal inlet which tends to be narrow, due to the weight of the larynx in the supine position, was opened by pulling the jaw forward using VLS. Impaction of piriform sinuses was suspected in these patients. In three patients (17.6%, 3/17), the NGT always advanced toward the trachea, due to anatomical reasons. The NGT could be advanced toward the piriform sinuses, but could not be inserted in the esophagus in another four patients (23.5%, 4/17), due to the arytenoid cartilage impaction. Despite the differences in patient status under general anesthesia and during CPR, the sites of impaction were relatively similar. Moreover, we perceived resistance in the middle or lower esophagus, and discontinued the insertion any further in four patients. This was probably due to the esophageal stenosis or hiatus hernia [17].

Previous studies have reported that complications associated with NGT insertion, such as wrong placements, pneumothorax, bleeding, and mucosal injury were more prone to occur in patients with obesity [18] and acute stroke [17], who experience difficult insertion. In our study, we focused on the laryngopharyngeal mucosal injury due to blind NGT insertion during CPR. A previous study reported that the most common regions of mucosal injury during NGT insertion were the arytenoid cartilage and piriform sinus [11]. Contrastingly, the most severe mucosal injuries occurred in the posterior wall of the pharynx (RPW), compared to other regions in both the Smooth and Dif groups in the present study. Previous studies included patients undergoing general anesthesia in an operating room [11,12]. In contrast, the present study included patients with CPOA who were undergoing continuous CPR; thus, their necks may have been hyperextended, and passive neck movements during CPR might be involved in RPW mucosal injury [19]. Moreover, we noticed sharp corners that were created when we pushed an NGT down; this impacted in the middle or lower esophagus, shown through the VLS image. These sharp corners might injure the RPW, in addition to injury from the tip of the NGT. We evaluated the NGT insertion number, in addition to the NGT insertion time. Both insertion time and insertion number showed strong positive correlations with laryngopharyngeal mucosal damage, with longer insertion time and increased insertion number resulting in more severe mucosal damage. In contract, little or no mucosal injury occurred in cases in which the NGT insertion was performed in one or two attempts or within 1 min. From these results, we believe that, for patients in whom the NGT cannot be blindly inserted smoothly, we should temporarily interrupt the procedure and switch to other methods, without any unnecessary persistence. This may minimize laryngopharyngeal mucosal damage associated with NGT insertion during CPR.

Several methods have been proposed for the insertion of NGTs in patients who are intubated [16,20]. It has been reported that VLS-assisted NGT insertion is effective [12,18,21]. On the other hand, Nasr Isfahani and Nasri Nasrabadi [18] have recently reported that NGT insertion using two digital methods is less time-consuming and has a higher success rate, compared with VLS-assisted insertion in intubated patients at the ED. Our study also revealed that in most patients undergoing resuscitation with CPOA, the NGT was blindly inserted smoothly. The indications for VLS-assisted NGT insertion are controversial. Our results indicated that VLS-assisted NGT insertion was useful in patients with impacted piriform sinuses or arytenoid cartilages, and with advances into the trachea. However, the VLS-assisted NGT insertion was not useful in patients in whom the NGT impacted in the middle or lower esophagus. From these results, in a case where blind NGT insertion is difficult, we should switch to VLS-assisted insertion or discontinue the insertion without persistence. We believe that blind NGT insertion performed within 1 min, or for a maximum of two or three times, might minimize laryngopharyngeal mucosal injury during the CPR at the ED.

The strengths of our study were the identification of locations where laryngopharyngeal mucosal injury was likely to occur due to NGT insertion and the demonstration that mucosal injury became more severe as the time and number of NGT insertions increased. Moreover, we have addressed the causes of patients with difficult NGT insertion during resuscitation. using recorded VLS videos. Our findings may contribute to the reduction in the risk of laryngopharyngeal mucosal injury from NGT insertion during resuscitation.

Our study has some limitations that need to be acknowledged. First, it was a single-center retrospective study, and the results for the Dif group were based on 17 patients, which is a small sample size. Hence, there is a possibility that potential biases might exist in retrospective analysis. We need further prospective randomized controlled trials or multiple-center studies based on these results. As most of the included patients died due to cardiopulmonary arrest, it was impossible to examine whether laryngopharyngeal injury caused hemorrhage due to anticoagulation and antiplatelet therapy. Laryngopharyngeal mucosal injury could have occurred during LMA insertion or during resuscitation by paramedics. Furthermore, because the patients had CPOA, we could not study the effects of muscle relaxation or sedative agents. Thus, further research is needed to clarify the optimal technique for NGT insertion at the ED.

## 5. Conclusions

The present study demonstrated that blind NGT insertion during CPR could be performed smoothly in most patients. However, the NGT cannot be inserted blindly in certain patients. The severity of laryngopharyngeal mucosal injury increased with the increase in NGT insertion time and insertion number. Whenever blind NGT insertion is difficult, we should switch to other methods, without any unnecessary persistence. VLS-assisted NGT insertion is one of the options for patients with difficult insertion. We believe that our findings are clinically applicable to patients undergoing CPR and to other intubated patients in the ED.

## Figures and Tables

**Table 1:** Patient characteristics and NGT insertion time and number.

Characteristic	Smooth (*n* = 67)	Dif (*n* = 17)	*p*-Value
Age (years)	77 (61.5–85)	77 (71–84)	0.5
Sex	Male 35, Female 32	Male 13, Female 4	0.12
Number of NGT insertions	1 (1–2)	8 (6–11)	0.0084
NGT insertion time	48.8 ± 4.0 s	Over 120 s (54.8 ± 3.0 s after switching VLS)	

NGT: nasogastric tube, Dif: Difficult group. Data are presented as median (interquartile range) and mean ± standard error.

**Table 2:** The injury score during Nasogastric tube insertion.

Location	Smooth (*n* = 67)	Dif (*n* = 17)	*p*-Value
RPW	0 (0–1)	2 (2–4)	0.0051
Px/VC	0 (0–1)	2 (1–3)	0.0074
Eg/VL	0 (0)	2 (0–3)	0.031
Total (RPW, Px, VC, Eg, VL)	0 (0–1)	7 (4–8)	0.0041

Data are presented as median (interquartile range). Eg: Epiglottis, Px: Pharynx, RPW: Retropharyngeal wall, VC: Vocal cords, VL: Vallecula.

**Table 3:** Simple regression analysis results.

Factor	Correlation Coefficient (*r*)	*n* = 84
Injury score, Age	0.11	
Injury score, Insertion time	0.79	*
Injury score, Insertion number	0.76	*
Age, Insertion time	0.13	
Age, Insertion number	0.18	
Insertion time, Insertion number	0.76	*

* *p* < 0.05, strong positive correlation (Pearson’s correlation coefficient values).

## Data Availability

The datasets used and analyzed during the current study are available from the corresponding author on reasonable request.

## Supplementary Materials

The following supporting information can be downloaded at: https://www.mdpi.com/article/10.3390/jcm13010261/s1, Video S1. A video showing the advancement of a nasogastric tube toward the trachea; Video S2. A video showing the advancement of a nasogastric tube toward the esophageal inlet. However, in this attempt, the tube could not be inserted into the esophagus; Video S3. A video showing failed nasogastric tube advancement through the middle or lower esophagus, due to perceived resistance during insertion; Video S4. A video demonstrating the sharp edges created when the nasogastric tube is bent due to resistance during insertion. These sharp corners attached to the retropharyngeal wall.
